# Recent Progress in the Management of Obesity: Advances and Insights from the Latest Research

**DOI:** 10.3390/nu17071225

**Published:** 2025-03-31

**Authors:** Javier Gómez-Ambrosi

**Affiliations:** 1 Metabolic Research Laboratory, Clínica Universidad de Navarra, 31008 Pamplona, Spain; jagomez@unav.es; 2 Centro de Investigación Biomédica en Red-Fisiopatología de la Obesidad y Nutrición (CIBEROBN), Instituto de Salud Carlos III, 31008 Pamplona, Spain; 3 Obesity and Adipobiology Group, Instituto de Investigación Sanitaria de Navarra (IdiSNA), 31008 Pamplona, Spain

## 1. Introduction

The field of obesity research continues to evolve rapidly, driven by new insights into metabolic regulation, behavioral interventions, and disparities in treatment outcomes [1,2,3]. This Special Issue, “Featured Articles on Nutrition and Obesity Management II” follows up on the previous edition by presenting cutting-edge research on obesity and its related comorbidities [4]. The ten articles featured in this Special Issue explore diverse aspects of obesity management, including pharmacological approaches, metabolic and physiological predictors, disparities in health outcomes, surgical interventions, and behavioral modifications. Together, they highlight the complexity of obesity and the necessity of a multifaceted approach to its treatment [5,6].

## 2. The Growing Burden of Obesity and the Need for Innovative Approaches

Obesity remains one of the most pressing public health challenges worldwide, contributing to an increased risk of metabolic disorders, cardiovascular diseases, and certain types of cancer [7]. Despite significant advancements in our understanding of the pathophysiology of obesity, effective long-term management remains elusive for many individuals [8,9]. The articles in this Special Issue underscore the need for personalized and multidisciplinary strategies, addressing pharmacological, behavioral, and surgical interventions while also considering socio-demographic disparities.

## 3. Advances in Pharmacological and Metabolic Interventions

One promising avenue in obesity treatment is pharmacological intervention [10,11]. Cicuéndez et al. (Contribution 1) explore a novel combination therapy using a dopamine receptor 2 agonist and a kappa opioid receptor antagonist, demonstrating synergistic effects in reducing weight by increasing thermogenic activity in rodents with diet-induced obesity. This study highlights the potential of targeting central nervous system pathways to enhance weight loss outcomes, opening doors for future clinical applications. Zhang et al. (Contribution 2) investigate the predictive roles of the basal metabolic rate (BMR) and skeletal muscle mass in lung function among patients with obesity-exacerbated asthma. Their study suggests that increased BMR and skeletal muscle mass may mediate the detrimental effects obesity has on spirometry in patients with asthma. Their findings underscore the importance of metabolic health for respiratory function, suggesting that personalized metabolic assessments could improve the management of obesity-related comorbidities.

## 4. Early-Life Influences and Long-Term Consequences

The impact of maternal and early-life nutrition on long-term obesity risk is another critical area of research [12,13]. Holt et al. (Contribution 3) examine how maternal high-fat diets and post-weaning high-carbohydrate diets contribute to increased rates of spontaneous hepatocellular carcinoma in aged mouse offspring. Their findings emphasize the need for targeted nutritional interventions during pregnancy and early childhood to mitigate future metabolic disorders. Similarly, Hernandez et al. (Contribution 6) provide valuable insights into how continuous glucose monitoring metrics in early and late pregnancy predict neonatal adiposity, particularly in mothers with obesity (as compared to normal weight counterparts) and despite following macronutrient-controlled eucaloric diets. This study underscores the complex interplay between maternal glucose regulation and fetal development, reinforcing the importance of metabolic monitoring during pregnancy.

## 5. Disparities in Cardiometabolic Impact and Potential Treatments in Adolescents with Obesity

The marked global increase in the prevalence of obesity in children and adolescents has intensified the research into potential treatments at early ages [14,15]. Understanding the disparities in obesity-related health outcomes is crucial for developing equitable treatment strategies [16,17]. Velasquez-Mieyer et al. (Contribution 4) analyze the cardiometabolic impact of adiposity among African American and Hispanic adolescents, revealing significant differences in metabolic risk factors. Their work highlights the need for culturally tailored interventions to address these ethnoracial disparities and improve health outcomes in different populations. Niechcial et al. (Contribution 9) provide a comprehensive review of current treatment perspectives for adolescents with obesity and type 2 diabetes. Their work synthesizes the latest evidence on pharmacological and behavioral interventions, advocating for an integrative approach to managing obesity in younger populations.

## 6. Surgical Interventions and Long-Term Outcomes

Bariatric surgery remains a highly effective intervention for severe obesity, with growing evidence on its metabolic benefits beyond weight loss [9,18]. Martínez-Montoro et al. (Contribution 7) investigate weight loss outcomes after sleeve gastrectomy in patients with metabolic dysfunction-associated steatotic liver disease. The authors find that the presence of steatohepatitis may be independently associated with lower weight loss after the surgery. Their study, based on liver biopsy data, provides crucial insights into how preoperative liver health influences post-surgical weight loss trajectories. On the other hand, Ozeki et al. (Contribution 8) further explore the long-term metabolic effects of laparoscopic sleeve gastrectomy in Japanese patients with obesity and diabetes. Their findings demonstrate sustained improvements in body composition and metabolic profiles three years post surgery, reinforcing the procedure’s efficacy as a long-term solution for obesity-related metabolic disorders. The reduction in fat mass and the maintenance of skeletal muscle mass are proposed as possible mediators of these long-term effects.

## 7. Behavioral and Lifestyle Interventions

Behavioral modifications remain a cornerstone of obesity management [19,20]. Parker et al. (Contribution 5) present a pilot randomized controlled trial testing a smartphone-delivered food attention retraining program in adolescent girls with overweight or obesity. Their findings suggest that digital interventions could play a crucial role in reshaping eating behaviors and reducing unhealthy food cravings in young populations. Finally, Jiménez-Ten Hoevel et al. (Contribution 10) explore the effects of chewing gum on satiety, appetite regulation, energy intake, and weight loss in a systematic review. Their analysis suggests that this simple behavioral strategy may contribute to reduced energy intake and improved weight management, though further research is needed to establish its long-term efficacy.

## 8. Conclusions

This second edition of “Featured Articles on Nutrition and Obesity Management” provides a comprehensive overview of recent advancements in obesity research. From novel pharmacological approaches and metabolic predictors to disparities in treatment outcomes and surgical interventions, the studies featured in this Special Issue reflect the multifaceted nature of obesity management [1]. As the field continues to evolve, integrating these diverse strategies will be essential in developing effective, personalized treatments that address both the biological and socio-environmental determinants of obesity. Future research should continue to refine these approaches, ensuring that obesity management strategies are inclusive, innovative, and effective for diverse populations [2].
